# Developing and Validating a Machine Learning Model to Predict Brain Injury in Preterm Infants Using Multisource Data from the Early Postnatal Period

**DOI:** 10.3390/children13060796

**Published:** 2026-06-09

**Authors:** Pu Xu, Ying Li, Ying Chen, Tongying Han, Peicen Zou, Qinglin Lu, Dongmiao Zhang, Jie Chen, Yajuan Wang

**Affiliations:** 1 Capital Institute of Pediatrics, Chinese Academy of Medical Sciences & Peking Union Medical College, Beijing 100020, China; xupuxp@126.com (P.X.); zou_peicen@sina.com (P.Z.); lql18845130328@163.com (Q.L.); 2 Department of Neonatology, Capital Center for Children’s Health, Capital Medical University, Capital Institute of Pediatrics, Beijing 100020, China; jizhenly@163.com (Y.L.); nhcy521@126.com (Y.C.); hanying6666@163.com (T.H.); dm_zhang2001@163.com (D.Z.); chenjief12@163.com (J.C.)

**Keywords:** preterm, brain injuries, neurodevelopmental impairment, machine learning, predictive model

## Abstract

Background: Moderate-to-severe preterm brain injury (PBI), including intraventricular hemorrhage (IVH) and periventricular leukomalacia (PVL), remains an important cause of adverse neurodevelopmental outcomes in preterm infants. Early risk stratification using routinely collected clinical data may help prioritize surveillance in vulnerable infants. Methods: We retrospectively included 318 preterm infants admitted between 2015 and 2024 as the development cohort. Thirty-three candidate predictors derived from perinatal factors, first laboratory tests within 24 h of admission, and selected early hospitalization variables were evaluated. Seven machine-learning algorithms were developed using stratified 10 × 5 nested cross-validation with prespecified preprocessing, class-balancing, and feature-selection procedures. Candidate models were compared primarily using the mean fold-level area under the receiver operating characteristic curve (AUROC). After model selection, the finalized LightGBM model was calibrated using Platt scaling, and its pooled out-of-fold (OOF) performance was summarized. Two prespecified thresholds (Youden and high-sensitivity) were used for risk stratification. A small independent temporal cohort of 35 infants was used for preliminary external validation. Results: PBI occurred in 62/318 infants (19.5%) in the development cohort and 6/35 infants (17.1%) in the temporal external cohort. During candidate-model comparison, LightGBM achieved the highest mean fold-level AUROC (0.768, 95% CI 0.708–0.825). The finalized 14-feature LightGBM model, evaluated using pooled OOF predictions after Platt calibration, yielded an AUROC of 0.747 (95% CI 0.679–0.811), a PR-AUC of 0.392, and a Brier score of 0.136. At the Youden threshold (0.18), sensitivity was approximately 0.70 and specificity approximately 0.85; at the high-sensitivity threshold (0.10), sensitivity was approximately 0.95 and specificity approximately 0.50. Key predictors included ventilation status and early physiologic and laboratory indicators. In the small temporal external cohort (*n* = 35), the AUROC was 0.897 (95% CI 0.672–1.000); however, this high point estimate should not be overinterpreted because of the limited sample size, wide confidence interval, and suboptimal calibration, and should therefore be considered preliminary. Conclusions: We developed an interpretable LightGBM model using routinely available early postnatal and early hospitalization data to support risk stratification for PBI in preterm infants. The model showed moderate internal discrimination and a positive net benefit across clinically relevant thresholds. Preliminary temporal external validation in a small cohort yielded highly uncertain estimates; larger multicenter studies are needed to confirm generalizability, refine calibration, and determine the most appropriate implementation strategy before routine clinical use.

## 1. Introduction

In recent years, although overall birth rates have declined, the rate of preterm birth remains high [1]. With advances in perinatal care, more preterm infants survive; however, the incidence of acute brain injuries such as intraventricular hemorrhage (IVH) and periventricular leukomalacia (PVL) remains substantial [2,3]. Among extremely preterm infants, IVH occurs in approximately 20–38%, and about 15% of those at 28–32 weeks develop IVH [4,5]. Despite continuous improvements in neonatal intensive care, these rates have not markedly decreased in recent decades [6,7]. Once preterm brain injury (PBI) occurs, even grade I-II IVH may have potential neurodevelopmental effects, whereas grade III or higher IVH drastically reduces the probability of survival without severe impairment [7]. Survivors commonly experience lasting sequelae [8,9,10]. However, early identification of high-risk infants remains challenging. Most IVH cases occur within 72 h after birth [11,12]. Before injury onset, vital signs and neurologic signs are often nonspecific. Effective treatments for PBI are limited. Even for hypoxic–ischemic encephalopathy (HIE), therapeutic hypothermia must be initiated within 6 h after birth [13,14]. Serial cranial ultrasound remains central to routine surveillance for brain injury in preterm infants; however, imaging-based follow-up primarily identifies injury once structural changes become radiologically apparent. A complementary risk-stratification approach based on routinely collected early clinical and laboratory data may help identify infants who warrant heightened surveillance between scheduled examinations or when imaging findings are not yet definitive. Therefore, precise early postnatal risk stratification of PBI using multisource clinical data is of clinical relevance.

Existing studies have developed various models to predict PBI, often reporting high discrimination [15,16]. However, most models have been evaluated only on retrospective single-center data with small samples, and their generalizability remains unknown [17]. Some models employ complex “black box” algorithms that are difficult to interpret in clinical practice [18,19,20].

In this study, using a nested cross-validation framework, we evaluated model discrimination and calibration while selecting a parsimonious set of key features. We predefined two risk thresholds to enable both general risk stratification and high-sensitivity screening, and we evaluated the potential clinical benefit of the model via decision curve analysis (DCA). We also performed SHAP interpretability analyses to assess the clinical plausibility of model outputs and developed a research-use online prototype to illustrate how calibrated risk estimates and individualized explanations could be presented in a surveillance-support workflow. Finally, we explored the relationship between PBI and neurodevelopmental impairment (NDI) at 6 months of age in a strictly exploratory analysis.

## 2. Materials and Methods

### 2.1. Data Collection and Processing

This single-center retrospective cohort study was conducted at the Capital Center for Children’s Health, Capital Medical University. The enrollment period for the modeling cohort was from August 2015 to June 2024. Inclusion criteria were: (1) preterm infants with gestational age ≤36 weeks; (2) admission to the neonatal department; and (3) complete data on key features and outcomes, or data that could be handled using the prespecified multiple-imputation framework. Exclusion criteria were: (1) presence of severe congenital malformations, chromosomal abnormalities, or inherited metabolic disorders; (2) missing core outcome data that could not be recovered; and (3) repeated hospitalizations, in which case only the first admission record was included. In addition, we collected 35 preterm infants admitted to the same neonatal department between July 2024 and April 2025 as an independent temporal external validation cohort. All data were handled under the same standards with an aligned and reproducible processing workflow. The study was approved by the Institutional Ethics Committee (Approval No.: SHERLLM2024043; Approval Date: [29 October 2025]), with informed consent waived due to the retrospective nature of the study and the use of de-identified data. Access to the study data was restricted to authorized investigators in accordance with institutional data-governance requirements.

### 2.2. Outcome Definition

All infants underwent regular cranial ultrasound screening, routinely within the first 3 days after birth, at approximately 1 week and 2 weeks of age, and weekly thereafter until discharge. When necessary, brain magnetic resonance imaging (MRI) was performed to confirm white matter injury. IVH and PVL were graded according to the Papile and de Vries classification systems. The primary outcome was PBI during neonatal hospitalization, defined as any brain injury meeting either of the following criteria: (1) IVH of Papile grade II or higher; (2) PVL of de Vries grade II or higher. The highest grade recorded in any imaging study during the hospital stay was used as the final outcome grade. In the external cohort, we assessed neurodevelopmental impairment (NDI) at a corrected age of 6 months using the Griffiths III developmental scales. NDI was defined as a developmental quotient below the 16th percentile (1 SD) in any domain or evidence of significant PVL sequelae on MRI at 6 months.

### 2.3. Candidate Features and Data Collection

Data were extracted from the electronic medical record system. After variable screening and data review, 33 candidate features were retained, including: (1) neonatal birth and admission information: sex, gestational age (weeks), and birth weight (g), among others; (2) maternal perinatal information: antenatal magnesium sulfate (MgSO_4_) administration, among others; (3) first laboratory tests within 24 h of admission: platelet count, lactate, albumin, white blood cell count, hemoglobin, and base excess, among others; (4) in-hospital complications and treatments: neonatal respiratory distress syndrome (NRDS), anemia of prematurity (AOP), surgical procedures during hospitalization (yes/no), and invasive mechanical ventilation days, among others. Because the predictor set included selected hospitalization variables, the model should be interpreted as an early postnatal risk-stratification tool during neonatal hospitalization rather than a purely antecedent or at-birth prediction model.

### 2.4. Data Preprocessing and Imputation

All data were transformed into an analyzable format. Missing data were handled using multiple imputation. Among the key laboratory features included in the final model, such as lactate and albumin, no missing data were observed. All imputation parameters for handling missingness were fitted strictly within the training data to avoid information leakage. For tree-based models, features were not standardized. For the logistic regression and multilayer perceptron (MLP), we performed z-score normalization within each training fold and applied the same transformations to the corresponding validation data. Given the class imbalance (~20% PBI incidence), we used class-balanced weights for PBI-positive and PBI-negative cases such that the total weight of positive samples was approximately equal to that of negatives. For the Balanced Random Forest, no additional weighting was applied to avoid over-correction.

### 2.5. Candidate Model Set and Hyperparameter Optimization

We considered seven machine-learning algorithms as candidate models: Light Gradient Boosting Machine (LightGBM), Balanced Random Forest, ExtraTrees, CatBoost, Explainable Boosting Machine (EBM), MLP, and regularized logistic regression. Model development and evaluation were performed using a 10 × 5 stratified nested cross-validation approach. We used Bayesian hyperparameter optimization within each training fold. All preprocessing steps and class-weighting were confined to the training portion of each fold. After tuning, we aggregated the optimal hyperparameters from the 10 outer folds to obtain a final recommended hyperparameter set for each model.

### 2.6. Feature Selection

To improve parsimony and generalizability, we conducted feature selection within the training cohort only using a prespecified framework based on permutation-importance ranking, top-k subset evaluation, and robustness assessment after correlation pruning. Using the final tuned models, we ranked candidate predictors by permutation importance and evaluated top-k feature subsets through cross-validation to identify an optimal feature set based on mean area under the receiver operating characteristic curve (AUROC). To assess robustness, we repeated the procedure after removing highly correlated predictors using an absolute Spearman correlation threshold of 0.85. Final subsets were selected from these candidate ranges with clinician review, balancing statistical performance with variable stability, clinical plausibility, routine availability, interpretability, and feasibility for bedside use.

### 2.7. Internal Validation and Model Comparison

Using the selected features and recommended hyperparameters, we trained each of the seven candidate models on the development cohort and evaluated their performance via nested cross-validation. We computed discrimination metrics (AUROC and PR-AUC) and calibration metrics (Brier score, calibration slope, intercept) for each model based on out-of-fold predictions. To assess potential clinical utility, we performed DCA comparing a strategy of intervening based on the model’s prediction with treat-all and treat-none strategies over a range of risk thresholds (0.05–0.30). Considering overall discrimination, calibration, and net benefit together, we selected the primary model for further study.

### 2.8. Primary Model Calibration and External Validation

After selecting the primary model, we calibrated the predicted probabilities using logistic regression calibration (Platt scaling). Using the development dataset, we prespecified two thresholds on the calibrated probability scale: a Youden threshold, defined as the probability that maximized the sum of sensitivity and specificity, and a high-sensitivity threshold, defined as the lowest probability achieving a sensitivity of at least 0.95.

The primary model was then fixed and applied to the external validation cohort. For each infant, the model generated a predicted probability of preterm brain injury. Model performance in the external cohort was evaluated using the AUROC, PR-AUC, Brier score, and calibration slope and intercept.

### 2.9. Model Interpretation and Visualization

To interpret the final model, we used Shapley additive explanations (SHAP). We generated global explanation plots, including a bar chart of mean absolute SHAP values and a SHAP beeswarm plot. In addition, we produced individual-case SHAP waterfall and force plots for representative infants.

### 2.10. Web Tool Construction

To illustrate how model outputs could be presented in a future NICU surveillance-support workflow, we built a research-use web prototype (https://pbi-app-rgexrpoidxuvm6btgacigu.streamlit.app/ [20 November 2025]). The prototype accepts the 14 model features, returns the calibrated predicted probability and risk category according to the predefined thresholds, and displays individualized SHAP waterfall and force plots. The tool does not store user-entered data, allows export of an HTML report, and is not intended for clinical diagnosis, treatment selection, or replacement of serial cranial ultrasound.

### 2.11. Exploratory Analysis of Long-Term Outcomes

We explored the relationship between PBI and NDI in the external cohort in a strictly exploratory, hypothesis-generating analysis. We calculated the risk ratio (RR), odds ratio (OR), and risk difference (RD) for NDI between infants with and without PBI, along with their 95% confidence intervals.

### 2.12. Statistical Software

All analyses were performed in Python 3.11. The main libraries included scikit-learn (1.7.2), lightgbm (4.6.0), catboost (1.2.8), shap (0.48.0), interpret (0.7.2), matplotlib (3.10.6), and others. For all stochastic procedures, we fixed random_state = 42.

### 2.13. Statistical Analysis

Continuous variables were summarized as mean ± standard deviation or median (IQR) and compared using appropriate parametric or nonparametric tests. Categorical variables were compared using the chi-square test or Fisher’s exact test. We reported effect sizes as Cohen’s d for continuous variables, Cliff’s δ for non-parametric comparisons, and ORs with 95% confidence intervals for categorical outcomes.

## 3. Results

### 3.1. Study Population and Analysis Workflow

**Figure 1:**
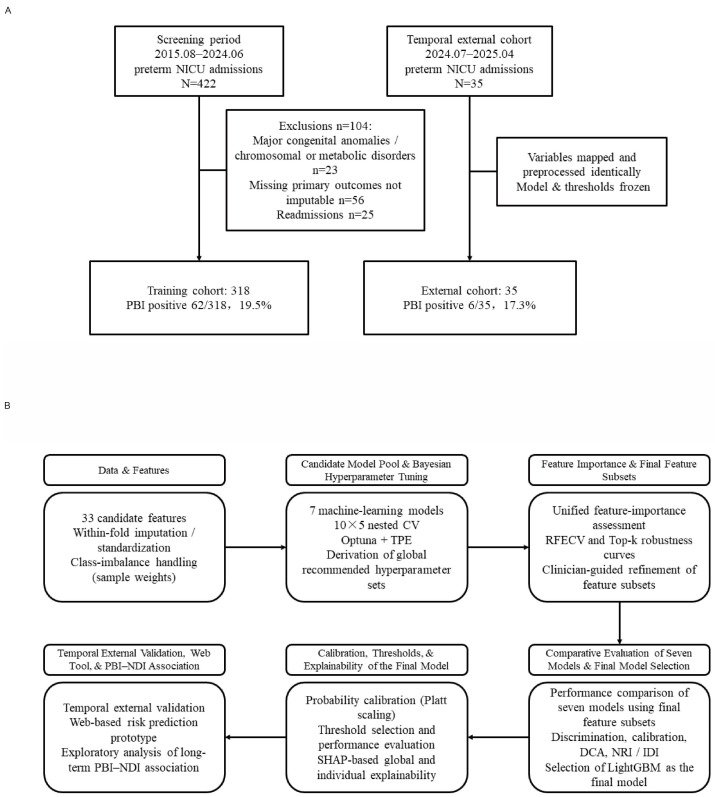
Flowchart and overall workflow. (**A**) Flow of participant inclusion and exclusion. (**B**) Overall workflow for the development, validation, and deployment of a machine learning model to predict PBI in preterm infants based on multisource data from the early postnatal period.

The final training cohort included 318 preterm infants, among whom 62 were PBI-positive, yielding a positivity rate of 19.5%; 42 met the IVH criterion, 27 met the PVL criterion, and 7 met both criteria. The independent temporal external validation cohort comprised 35 infants, including 6 PBI-positive cases, with a positivity rate of 17.1%. The overall workflow for cohort construction, feature processing, model development, and validation is summarized in Figure 1.

### 3.2. Univariable Analysis

Between-group comparisons of demographic and clinical characteristics for PBI-positive and PBI-negative infants in the training cohort are shown in Table 1. The three most markedly different variables between the PBI and non-PBI groups were albumin, lactate dehydrogenase (LDH), and the 1-min Apgar score. Several indicators of immaturity and early illness severity differed between groups.

### 3.3. Feature Selection Results

After training each model on the full feature set and recommended hyperparameters, we examined the impact of different numbers of features on model performance. Considering both performance and stability, the final feature subset sizes for the various models ranged from 10–14 features. Once finalized, these reduced feature subsets were fixed and used in subsequent model training and testing.

### 3.4. Model Performance Comparison and Primary Model Selection

Using the selected feature subsets and tuned hyperparameters, we compared the seven candidate models (Table 2). The mean fold-level AUROC ranged from 0.700 to 0.768, with LightGBM achieving the highest value (0.768, 95% CI 0.709–0.825), followed by ExtraTrees (0.744) and EBM (0.737). Mean PR-AUC values ranged from 0.348 to 0.409. Brier scores were similar (0.139–0.148), whereas calibration slopes and intercepts indicated some between-model differences in probability calibration. Given its overall performance, stable feature reduction, and deployability, LightGBM was selected as the primary model for subsequent analyses.

### 3.5. Calibration and Interpretability of the Primary Model

**Figure 2:**
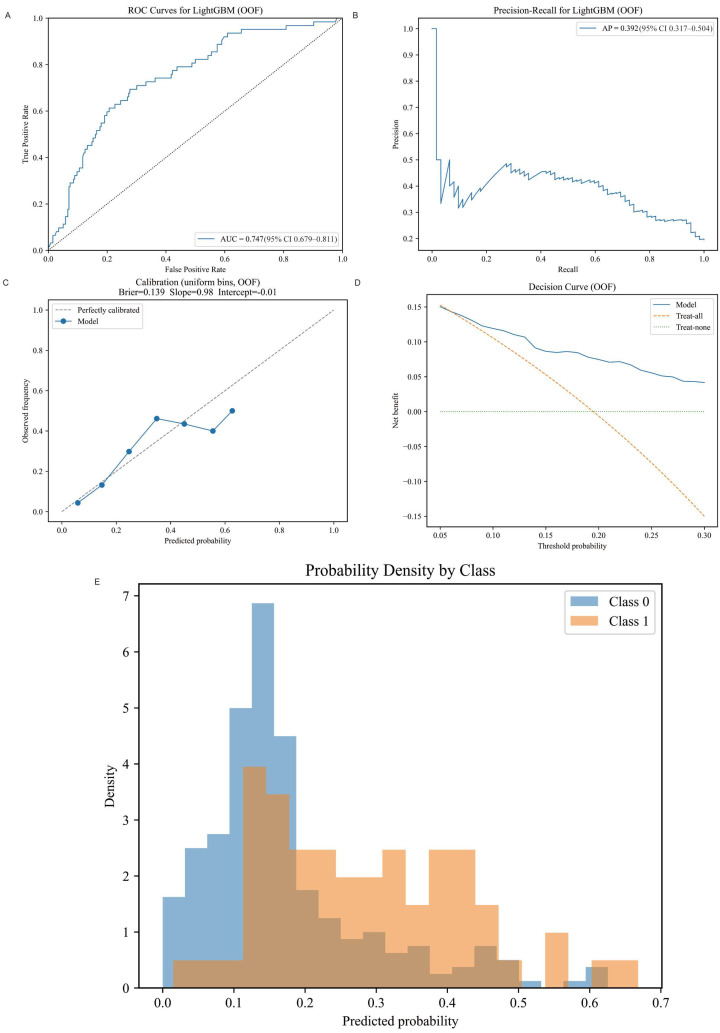
Performance evaluation of LightGBM model. (**A**) AUROC of LightGBM model. (**B**) PR-AUC of LightGBM model. (**C**) Calibration curve of LightGBM model. (**D**) DCA of LightGBM model. (**E**) Distribution of predicted probabilities from LightGBM model.

Internal validation of the finalized LightGBM model is summarized in Figure 2. Using pooled outer-fold out-of-fold predictions from the finalized LightGBM model after Platt calibration, discrimination remained moderate (AUROC 0.747, 95% CI 0.679–0.811; PR-AUC 0.392) with acceptable overall calibration (Brier score 0.136; calibration slope 0.98 and intercept −0.01). DCA showed positive net benefit compared with “treat none” and “treat all” strategies, particularly at threshold probabilities around 0.10–0.20. Predicted risk distributions showed clear separation between most infants without PBI and those with PBI. For practical risk stratification, we used a Youden threshold of 0.18 (sensitivity 70%, specificity 85%). For screening-focused scenarios, a high-sensitivity threshold of 0.10 increased sensitivity to approximately 95% but reduced specificity to about 50% and yielded a positive predictive value below 30%, indicating that this threshold may be more suitable for prioritized surveillance than for triggering high-cost interventions.

**Figure 3:**
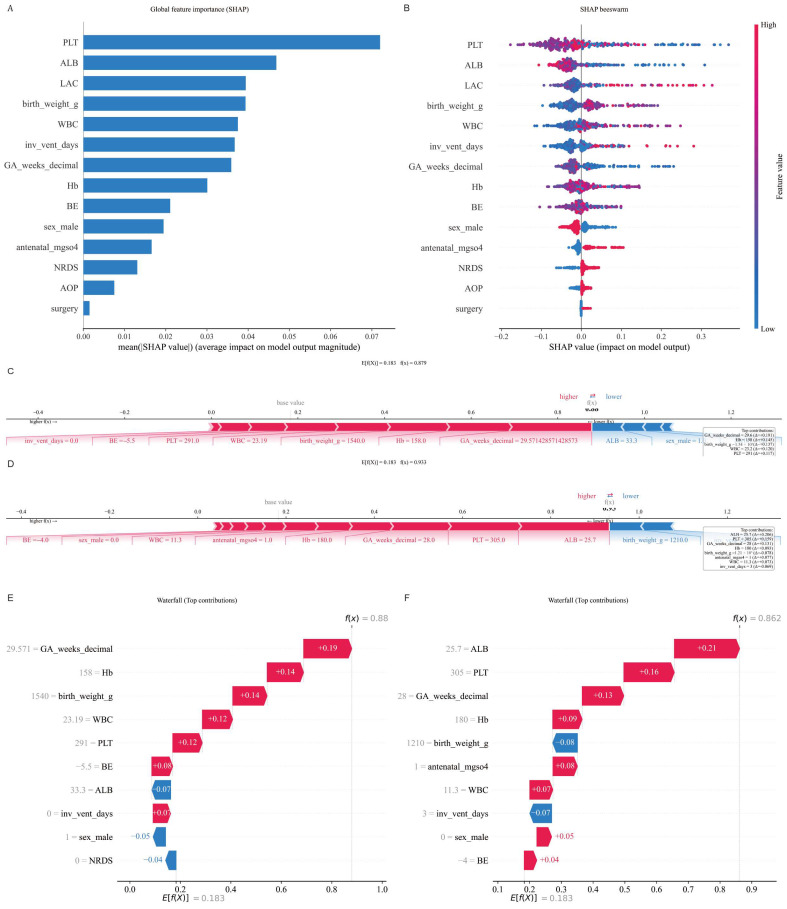
Global and individual SHAP for the LightGBM model. (**A**) SHAP feature importance bar plot. (**B**) SHAP beeswarm plot. (**C**,**D**) SHAP Force plots. (**E**,**F**) SHAP waterfall plots.

Global and individual-level interpretability is shown in Figure 3. The SHAP feature-importance bar plot and beeswarm plot indicated that platelet count, lactate, and gestational age were the most influential predictors. Variables reflecting early respiratory support and early hematologic status also contributed meaningfully to model outputs. To further illustrate clinical interpretability at the individual level, we generated SHAP explanation plots for representative cases, including waterfall plots and force plots. Overall, these interpretability findings align with known clinical risk factors for preterm brain injury and may help clinicians understand why a given infant is classified as high risk, thereby supporting closer review and surveillance prioritization.

### 3.6. Temporal External Validation

**Figure 4:**
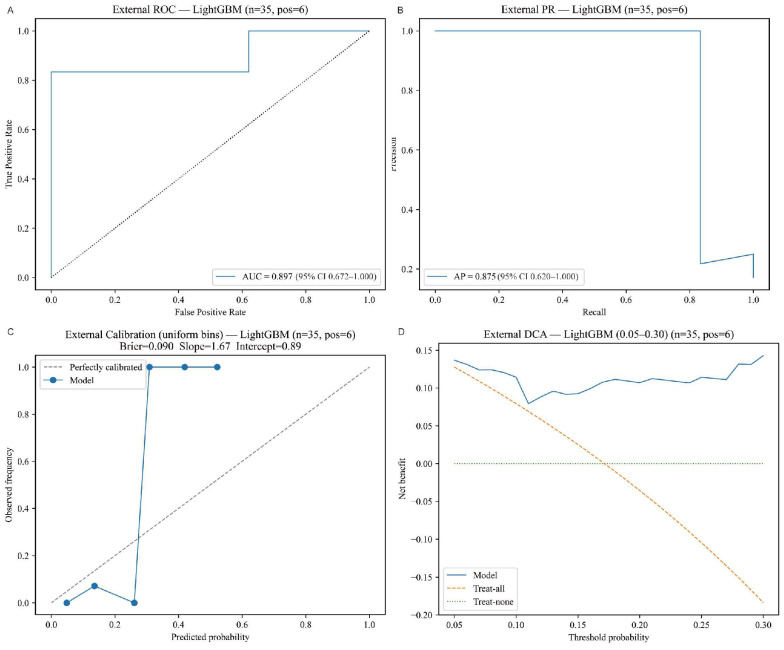
External validation results. (**A**) AUROC for the external validation set. (**B**) PR-AUC for the external validation set. (**C**) Calibration curve for the external validation set. (**D**) DCA for the external validation set.

We validated the finalized LightGBM model in the independent temporal external validation cohort. Discrimination and calibration performance are shown in Figure 4. In this independent sample, the AUROC was approximately 0.897 (95% CI 0.672–1.000), and the PR-AUC was approximately 0.875 (95% CI 0.620–1.000); however, these point estimates should not be overinterpreted because the external cohort was very small and the confidence intervals were wide. Given the very small external sample size (*n* = 35), the 95% CIs were extremely wide and encompassed the AUROC estimates from internal validation, suggesting that the apparently higher external performance likely reflects small-sample variation rather than stronger generalizability. In addition, the calibration curve showed substantial deviation in the external dataset, indicating that external probability estimates require further validation and potential recalibration. Overall, the model’s predicted probabilities tended to underestimate the observed event rates, and further validation in larger external cohorts is required.

### 3.7. Clinical Application Prototype

**Figure 5:**
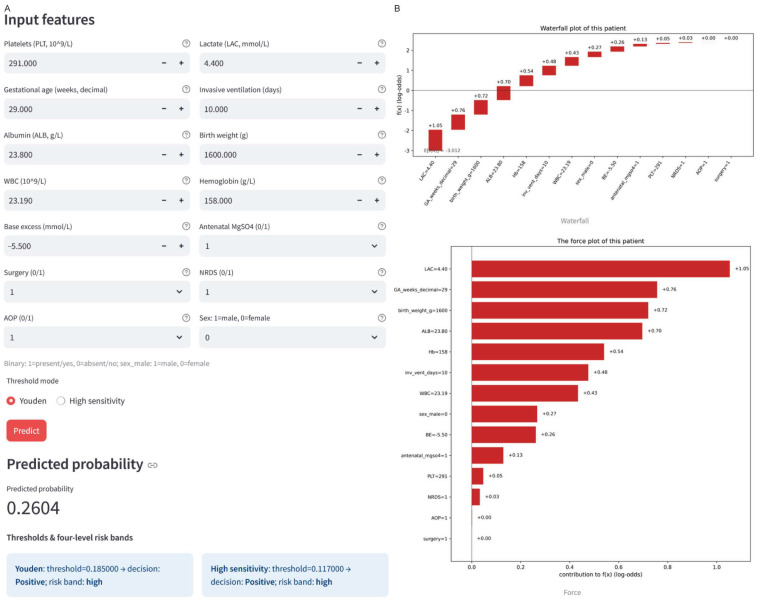
Web prototype interface for PBI risk prediction. (**A**) Input panel for single-case prediction using the 14 final features, with display of calibrated predicted PBI risk and risk stratification based on the predefined Youden and high-sensitivity thresholds. (**B**) SHAP waterfall and force plots illustrating feature contributions to the individual prediction.

To facilitate clinical translation, we developed a web-based application prototype. Figure 5 presents a screenshot of the interface. The tool provides input fields for the 14 features and, upon data entry, returns the predicted PBI risk along with the risk category (“low,” “medium,” or “high”) based on the two thresholds. It also displays individualized SHAP plots that explain the prediction for that infant. These capabilities allow clinicians to not only receive a risk estimate but also understand which factors are driving the risk for a given patient. The current prototype is intended for research and quality-control purposes only and should not be used as a stand-alone diagnostic tool.

### 3.8. Association Between Early PBI and Long-Term Outcomes

In the external cohort, 3 of 6 infants with PBI developed NDI (50%). PBI was associated with a higher risk of NDI; however, effect estimates were highly imprecise because of the very small sample size and sparse outcomes, and therefore should be interpreted cautiously (Supplementary Table S1). This finding should be considered hypothesis-generating only, and larger studies with longer follow-ups are needed before any inference regarding the magnitude of association can be made.

## 4. Discussion

In this study, we developed and internally evaluated an interpretable machine learning model for PBI risk stratification in preterm infants. Using 14 routinely available early-life and early hospitalization features, our LightGBM model achieved moderate discrimination in internal testing. We predefined two risk thresholds (a Youden threshold and a high-sensitivity threshold) to accommodate both general risk stratification and screening scenarios. DCA indicated that model-guided decisions would offer net clinical benefit across a range of threshold probabilities. SHAP analysis demonstrated that the most important features in the model, such as platelet count, lactate, and gestational age, were highly consistent with known risk factors for preterm brain injury, thereby enhancing the model’s clinical interpretability. In the external cohort, the AUROC point estimate was high, but this result should be interpreted cautiously because of the very small sample size, wide confidence intervals, and suboptimal calibration. We also observed a trend toward higher 6-month NDI incidence among infants with PBI; however, because of the very small sample and sparse outcomes, these estimates were highly unstable and should be interpreted with extreme caution. Overall, our results suggest that a model built on multisource clinical data may support early postnatal risk stratification and surveillance prioritization for severe brain injury in preterm infants in the NICU.

Several predictive models for PBI in preterm infants have been reported. Traditional logistic regression models can achieve moderate accuracy (around 0.73 AUC), but machine learning approaches in this domain have shown potential for higher performance or adaptability [21]. For example, a model using only four variables achieved an external AUROC above 0.85, and incorporating dynamic first-week data significantly improved predictions in another study [15,22]. Our model integrates information spanning the neonatal period, including perinatal conditions at birth, early lab results after admission, and major complications and interventions. As a result, it attained a moderate-to-high level of discrimination while relying on 14 features. DCA confirmed that model-based risk stratification yields a net benefit, in line with other neonatal models that demonstrated clinical utility via DCA [23]. However, direct comparison of AUROCs across studies should be interpreted cautiously because populations, outcome definitions, predictor sets, and case-mix differ substantially. More broadly, recent clinical machine-learning studies have increasingly emphasized that useful prediction models should not be judged by discrimination alone, but also by how risk estimates can be translated into operational pathways for monitoring, escalation, and clinical decision support [24].

External validation is essential yet challenging for predictive models. Many published IVH risk models for preterm infants have not been validated on independent cohorts [17,25]. In the temporal external cohort, the LightGBM model achieved an AUROC of 0.90 (95% CI 0.67–1.00), but its calibration was poor, likely due to differences in case mix or the small sample size [26]. Therefore, despite a numerically high point estimate on initial external testing, further evaluation in larger multicenter cohorts is needed to confirm generalizability and to recalibrate the model for new populations if required.

The features identified by our model align with known mechanisms of brain injury in preterm infants. Fragile cerebral vasculature and limited autoregulation make these infants susceptible to hemorrhage or ischemia under hypotensive and hypoxic conditions [27,28]. Elevated lactate with metabolic acidosis is associated with increased IVH risk [16]. Thrombocytopenia and coagulation abnormalities increase bleeding tendency and have been reported as significant risk factors for IVH [7,9]. Low albumin, often accompanying severe inflammation or poor nutrition, may compromise the vascular endothelium; correspondingly, hypoalbuminemia has been linked to higher risk of brain injury [8].

Model transparency is increasingly emphasized in clinical machine learning. Recent studies have used SHAP to interpret neonatal risk models [29]. In our study, SHAP analysis allowed us to explain individual predictions and verify that the model’s reasoning aligns with clinical knowledge. By contrast, some deep learning approaches can achieve high accuracy in detecting IVH from cranial ultrasound images but function as “black boxes” with limited interpretability, which may impede clinical adoption [19,27,30]. In practice, such explanations may help clinicians identify whether a high-risk prediction is primarily driven by physiological stress, hematologic vulnerability, or respiratory support burden, thereby facilitating more focused review rather than dictating specific treatment decisions. From a practical standpoint, these explanations may help clinicians review the risk dimensions underlying a high-risk estimate, such as physiologic instability, hematologic vulnerability, inflammatory status, or respiratory support burden, and may support consideration of closer cranial ultrasound surveillance or multidisciplinary reassessment when clinically appropriate.

An important practical consideration is that this model is not intended to replace cranial ultrasound, MRI, or clinical judgment, but rather to serve as part of the early PBI risk-assessment workflow in the NICU. Serial cranial ultrasound remains an important imaging modality for detecting IVH and PVL, whereas our LightGBM model may provide complementary risk information when imaging findings are not yet definitive. The model generates an individualized predicted probability of PBI and stratifies infants according to prespecified thresholds. The high-sensitivity threshold of 0.10, corresponding to approximately 95% sensitivity, may be suitable for screening-oriented scenarios to help identify as many potentially high-risk infants as possible. The Youden threshold of 0.18, corresponding to approximately 70% sensitivity and 85% specificity, may be more suitable for risk stratification when a relative balance between sensitivity and specificity is desired. For infants whose predicted risk reaches the high-sensitivity threshold but remains below the Youden threshold, the model output may prompt the clinical team to maintain heightened vigilance, intensify observation according to clinical changes, and pay closer attention to subtle or evolving imaging abnormalities during routine cranial ultrasound follow-up. For infants whose predicted risk reaches or exceeds the Youden threshold, the clinical team may further review vital signs, respiratory support status, hematologic status, and infection- or inflammation-related indicators, and may consider earlier or more frequent cranial ultrasound reassessment, repeat laboratory testing when appropriate, and earlier multidisciplinary evaluation. In addition, the SHAP waterfall and force plots in the web prototype display the contribution of each feature to the individual prediction, helping clinicians understand the potential sources of risk. For example, a high contribution from white blood cell count may prompt attention to infection or inflammation; lactate or BE may prompt review of circulatory perfusion, metabolic acidosis, or tissue hypoxia; hemoglobin or anemia of prematurity may prompt attention to anemia and oxygen-carrying capacity; and invasive ventilation days may reflect respiratory support burden and overall illness severity. Therefore, the main incremental value of the current model is to work alongside serial cranial ultrasound as an auxiliary tool for structured risk review and surveillance prioritization.

Our exploratory follow-up analysis suggests that neonatal brain injury may adversely affect later neurodevelopment. In our external sample, about half of the infants with PBI developed NDI by 6 months, versus a lower proportion among those without PBI. This observation is consistent with prior evidence that early brain injuries in preterm infants significantly increase the risk of subsequent neurodevelopmental impairments [7,10]. In anticipation of such long-term impacts, other groups have begun developing models to predict neurodevelopmental outcomes in preterm populations. For example, an ensemble model using multicenter data was able to predict 2-year cognitive delay in extremely preterm infants after feature selection with the Boruta algorithm [31]. However, Assessment of NDI using the Griffiths III developmental scales at a corrected age of 6 months is relatively early. Transient developmental delays are common at this age, whereas mild-to-moderate cognitive and behavioral deficits often become more apparent at 18–24 months. Therefore, our analysis should be regarded as a preliminary observation of early developmental milestone achievement.

This study has several innovations. We systematically compared multiple algorithms to identify the optimal model; used nested cross-validation to tune and validate performance without overfitting; and employed a feature selection strategy to build a simpler model without sacrificing accuracy. We also incorporated SHAP analysis to enhance model transparency, evaluated the model on a temporally independent cohort, included preliminary exploratory 6-month outcome descriptions, and developed a research-use web prototype to demonstrate how calibrated risk estimates and individualized explanations could be displayed.

Nonetheless, this study has several limitations. First, it was a single-center retrospective study with a limited sample size, which may introduce selection bias and limit generalizability. Second, the external validation cohort contained very few infants meeting the exploratory 6-month developmental endpoint, resulting in low statistical power; moreover, corrected-age 6-month Griffiths III assessment mainly reflects early developmental milestone achievement and requires longer-term follow-up at 18–24 months or later. Third, our model is based on static clinical features available in early life and does not incorporate dynamic changes or additional data modalities, which limits its ability to adapt to a patient’s evolving condition in real time. Moreover, because selected cumulative hospitalization variables were included, the model may partly reflect exposure accrued during hospitalization rather than purely antecedent risk. Therefore, the model should be interpreted primarily as an early in-hospital risk-stratification tool, and future studies should evaluate more strictly time-anchored variables such as mechanical ventilation status within 24 h of life or before cranial ultrasound. In addition, although missing data were handled using multiple imputation, residual uncertainty related to the retrospective missing-data structure cannot be completely excluded. Finally, the model’s performance and calibration should be further evaluated in larger, multicenter populations to ensure robustness across different hospitals and patient groups.

## 5. Conclusions

The LightGBM machine learning model developed in this study, based on routinely available early-life and early hospitalization clinical data, can estimate the risk of severe brain injury during the neonatal period in preterm infants with reasonable accuracy. Using 14 features, the model achieved moderate discriminative performance and acceptable overall calibration, with robustness assessed through nested cross-validation. Risk thresholds can be flexibly adjusted according to different application scenarios to balance sensitivity and specificity. The model’s key features are consistent with clinical experience, and SHAP-based analysis has increased the transparency of model decisions, facilitating clinical understanding and trust. Preliminary temporal external validation in a small independent dataset (*n* = 35) yielded highly uncertain estimates, and further evaluation and calibration refinement in larger multicenter populations are required. This model has the potential to serve as an early warning and risk-stratification tool for brain injury in preterm infants, assisting clinicians in identifying high-risk infants at an early stage and prioritizing surveillance and clinical review. Future multicenter studies are needed to further validate the model’s generalizability, continuously refine its functionality by incorporating additional data modalities, and strengthen follow-up research to clarify the causal relationship between brain injury in preterm infants and long-term neurodevelopmental impairment, thereby guiding strategies to improve long-term outcomes in this vulnerable population.

## Figures and Tables

**Table 1:** Statistics of characteristics between the PBI and non-PBI groups.

Characteristics	PBI	Non-PBI	OR (95% CI)/Cliff’s δ	*p* Value
Albumin (ALB)	27.50 [25.30, 30.30]	30.00 [27.77, 32.60]	−0.33	<0.001
Lactate Dehydrogenase (LDH)	571.00 [453.00, 791.00]	471.00 [395.00, 618.00]	0.27	0.001
Apgar 1 min	8.00 [7.00, 9.00]	9.00 [8.00, 10.00]	−0.25	0.002
Blood transfusion	46/62 (74.2%)	135/256 (52.7%)	2.53 (1.37–4.66)	0.002
Hydroxybutyrate Dehydrogenase (HBDH)	393.00 [312.00, 552.00]	343.50 [273.00, 447.25]	0.25	0.003
Anemia of Prematurity (AOP)	55/62 (88.7%)	180/256 (70.3%)	3.14 (1.40–7.04)	0.003
Surgery	14/62 (22.6%)	24/256 (9.4%)	2.84 (1.38–5.82)	0.004
Invasive ventilation days	3.00 [0.00, 7.00]	1.00 [0.00, 4.00]	0.22	0.006
Platelet (PLT)	172.00 [137.75, 266.75]	221.00 [179.00, 259.00]	−0.20	0.013
Sepsis	34/62 (54.8%)	97/256 (37.9%)	1.98 (1.14–3.45)	0.015
Apgar 5 min	9.00 [9.00, 10.00]	10.00 [9.00, 10.00]	−0.17	0.027
Intrauterine Growth Restriction (IUGR)	10/62 (16.1%)	20/254 (7.9%)	2.29 (1.03–5.10)	0.047
Lactic Acid (LAC)	2.15 [1.55, 4.33]	2.00 [1.45, 2.60]	0.16	0.050

**Table 2:** Comparison of performance evaluation metrics of 7 models.

Model	ROC-AUC (95% CI)	PR-AUC (95% CI)	Brier	Cal. Slope	Cal. Intercept
LightGBM	0.768 (0.708–0.825)	0.400 (0.327–0.513)	0.139	1.143	0.206
ExtraTrees	0.744 (0.669–0.813)	0.409 (0.331–0.533)	0.142	0.935	−0.091
EBM	0.737 (0.657–0.806)	0.385 (0.312–0.510)	0.141	1.063	0.148
Balanced Random Forest	0.725 (0.654–0.792)	0.359 (0.292–0.463)	0.144	0.782	−0.261
MLP	0.724 (0.653–0.793)	0.380 (0.308–0.500)	0.143	0.541	−0.623
CatBoost	0.713 (0.636–0.784)	0.380 (0.300–0.502)	0.142	1.078	0.155
Logistic regression	0.699 (0.623–0.768)	0.348 (0.272–0.452)	0.148	1.141	0.175

## Data Availability

The clinical data are not publicly available because of privacy and institutional restrictions. Researchers interested in the data for academic purposes may contact the corresponding author. The code for the research-use web application is available at https://github.com/lyrperciver/PBI-APP (20 November 2025).

## Supplementary Materials

The following supporting information can be downloaded at: https://www.mdpi.com/article/10.3390/children13060796/s1, Table S1: Association between early perinatal brain injury (PBI) during initial hospitalization and neurodevelopmental impairment (NDI) at 6 months in the temporal external validation cohort (*n* = 35).

## Abbreviations

ALB	Albumin
AOP	Anemia of prematurity
AP	Average precision
AUROC	Area under the receiver operating characteristic curve
BE	Base excess
BPD	Bronchopulmonary dysplasia
CI	Confidence interval
DCA	Decision curve analysis
DWI	Diffusion-weighted imaging
EBM	Explainable boosting machine
HBDH	Hydroxybutyrate dehydrogenase
HIE	Hypoxic–ischemic encephalopathy
Hb	Hemoglobin
IQR	Interquartile range
IUGR	Intrauterine growth restriction
LDH	Lactate dehydrogenase
LAC	Lactic acid
LASSO	Least absolute shrinkage and selection operator
LightGBM	Light Gradient Boosting Machine
ML	Machine learning
MLP	Multilayer perceptron
MRI	Magnetic resonance imaging
NDI	Neurodevelopmental impairment
NEC	Necrotizing enterocolitis
NICU	Neonatal intensive care unit
NRI	Net reclassification improvement
NRDS	Neonatal respiratory distress syndrome
OOF	Out-of-fold
OR	Odds ratio
PBI	Preterm brain injury
PLT	Platelet
PR-AUC	Area under the precision-recall curve
PROM	Premature rupture of membranes
PS	Pulmonary surfactant
PVL	Periventricular leukomalacia
RD	Risk difference
ROP	Retinopathy of prematurity
RR	Risk ratio
SD	Standard deviation
SHAP	Shapley additive explanations
T1WI	T1-weighted imaging
T2WI	T2-weighted imaging
TPE	Tree-structured Parzen Estimator
VLBWI	Very low birth weight infant
WBC	White blood cell count
