# Supplementary material for: Developing and Validating a Machine Learning Model to Predict Brain Injury in Preterm Infants Using Multisource Data from the Early Postnatal Period
Source: Children (Basel). 2026 Jun 9;13(6):796. doi: 10.3390/children13060796 (PMC13297451; doi:10.3390/children13060796)
Supplement: Supplementary file 1 [file children-13-00796-s001.zip › children-4368508-supplementary.pdf]

Supplementary Material

1. Supplementary Table S1. Association between early perinatal brain injury (PBI) during initial hospitalization and neurodevelopmental impairment (NDI) at 6 months in the temporal external validation cohort (n = 35)

Supplementary Table S1 Association between early perinatal brain injury (PBI) during initial hospitalization and neurodevelopmental impairment (NDI) at 6 months in the temporal external validation cohort (*n* = 35)

PBI status	Infants, n	NDI cases, n	NDI incidence, %	Risk ratio RR (95% CI)¹	Odds ratio OR (95% CI)¹	Risk difference RD (95% CI)¹	Chi-square test P value²	Fisher's exact test P value²	Phi coefficient²
PBI = 0	29	0	0	Reference	Reference	Reference	Reference	Reference	Reference
PBI = 1	6	3	50	30.00 (1.74–516.95)	59.00 (2.49–1395.74)	0.48 (0.11–0.86)	0.00007	0.0031	0.67

Effect estimates are for PBI-positive vs PBI-negative infants and were calculated using Haldane–Anscombe continuity correction; RR, risk ratio; OR, odds ratio; RD, risk difference.
